# A Novel Intelligent Two-Way Communication System for Remote Heart Failure Medication Uptitration (the CardioCoach Study): Randomized Controlled Feasibility Trial

**DOI:** 10.2196/cardio.9153

**Published:** 2018-04-04

**Authors:** Christophe JP Smeets, Valerie Storms, Pieter M Vandervoort, Pauline Dreesen, Julie Vranken, Marita Houbrechts, Hanne Goris, Lars Grieten, Paul Dendale

**Affiliations:** ^1^ Department of Cardiology Ziekenhuis Oost-Limburg Genk Belgium; ^2^ Future Health Department Ziekenhuis Oost-Limburg Genk Belgium; ^3^ Mobile Health Unit Faculty of Medicine and Life Sciences Hasselt University Hasselt Belgium; ^4^ Biomedical Research Institute Faculty of Medicine and Life Sciences Hasselt University Hasselt Belgium; ^5^ Department of Cardiology Jessa Ziekenhuis Hasselt Belgium

**Keywords:** heart failure, telemedicine, clinical decision support, drug monitoring, drug utilization, call centers

## Abstract

**Background:**

European Society of Cardiology guidelines for the treatment of heart failure (HF) prescribe uptitration of angiotensin-converting enzyme inhibitors (ACE-I) and β-blockers to the maximum-tolerated, evidence-based dose. Although HF prognosis can drastically improve when correctly implementing these guidelines, studies have shown that they are insufficiently implemented in clinical practice.

**Objective:**

The aim of this study was to verify whether supplementing the usual care with the CardioCoach follow-up tool is feasible and safe, and whether the tool is more efficient in implementing the guideline recommendations for β-blocker and ACE-I.

**Methods:**

A total of 25 HF patients were randomly assigned to either the usual care control group (n=10) or CardioCoach intervention group (n=15), and observed for 6 months. The CardioCoach follow-up tool is a two-way communication platform with decision support algorithms for semiautomatic remote medication uptitration. Remote monitoring sensors automatically transmit patient’s blood pressure, heart rate, and weight on a daily basis.

**Results:**

Patients’ satisfaction and adherence for medication intake (10,018/10,825, 92.55%) and vital sign measurements (4504/4758, 94.66%) were excellent. However, the number of technical issues that arose was large, with 831 phone contacts (median 41, IQR 32-65) in total. The semiautomatic remote uptitration was safe, as there were no adverse events and no false positive uptitration proposals. Although no significant differences were found between both groups, a higher number of patients were on guideline-recommended medication dose in both groups compared with previous reports.

**Conclusions:**

The CardioCoach follow-up tool for remote uptitration is feasible and safe and was found to be efficient in facilitating information exchange between care providers, with high patient satisfaction and adherence.

**Trial Registration:**

ClinicalTrials.gov NCT03294811; https://clinicaltrials.gov/ct2/show/NCT03294811 (Archived by WebCite at http://www.webcitation.org/6xLiWVsgM)

## Introduction

Heart failure (HF) is a major health problem affecting more than 10% in the elderly over the age of 70 years [1-5]. Mortality rates are high, with only 50% of patients surviving up to 5 years after first diagnosis. Hospitalization rates are even higher with 1-year hospitalization rates of approximately 40% and a readmission rate of 30% to 45% within 6 months after initial admission [6-9]. These high (re)admission rates put a large burden on the current health care system [10-12].

Improvements in treatment strategies have reduced mortality and (re)hospitalization rates. In 2016, the updated guidelines of the European Society of Cardiology (ESC) concerning the diagnosis and treatment of acute and chronic HF with reduced ejection fraction were published [1]. These guidelines prescribe uptitration of angiotensin-converting enzyme inhibitors (ACE-I) and β-blockers to the maximum-tolerated, evidence-based dose in function of a patient’s weight, blood pressure, heart beat, and kidney function. There is strong evidence that adherence to guidelines and optimal drug treatment leads to a better clinical outcome and reduced mortality and (re)hospitalizations [1]. HF disease management programs are widely used to facilitate the implementation of guideline-recommended treatment strategies [13-17]. Unfortunately, studies have proven that they are still insufficiently implemented in practice [15,18-20].

The addition of remote monitoring combined with integrated clinical decision support in this aspect could provide added value for both the health care provider and the patient. Remote monitoring of vital parameters and other patient information could allow care givers to evaluate and adjust patients’ medication schemes remotely according to ESC guidelines [21]. Remote β-blocker uptitration based on patient’s self-collected physiologic data transmitted by phone has been previously studied and showed a positive impact on β-blocker use [21-23]. The IN-TOUCH trial was one of the first studies to investigate the value of a decision support algorithm for medication uptitration in addition to remote monitoring (ie, weight, blood pressure, and electrocardiogram) compared with remote monitoring alone. However, this study lacked a usual care control group and could not show differences in clinical outcome [24,25]. Kropf et al also developed a remote monitoring strategy with integrated clinical decision support, but the algorithm was only retrospectively analyzed with existing remote monitoring datasets [26]. The aim of Kropf et al was to prospectively study this strategy in a large-scale randomized trial, but unfortunately, the trial was stopped and no results are available. Therefore, the CardioCoach study is the first to study the feasibility of remote monitoring with integrated decision support on HF drug treatment optimization.

The CardioCoach study combines a two-way communication platform with decision support algorithms together with remote monitoring sensors for active medication uptitration. The study will verify whether supplementing the guideline-driven usual care with this two-way communication platform can implement the guideline recommendations for β-blocker and ACE-I more efficiently. This paper focusses on the feasibility of the communication platform for adjusting HF medication remotely and for detecting early deterioration by monitoring blood pressure, heart rate, and weight changes. Patient’s vital measurements and therapy adherence were actively encouraged by the smartphone app and were evaluated together with the patient’s satisfaction of the CardioCoach tool.

## Methods

### Study Design

This is a prospective single-center randomized control feasibility trial conducted in a Belgian tertiary care center (Jessa hospital, Hasselt, Belgium) with a specialized HF disease clinic. Newly diagnosed patients with HF and initiation of β-blocker and/or ACE-I therapy or patients with known HF but on suboptimal dosage of β-blocker and/or ACE-I therapy were approached. Upon inclusion, block randomization was used to divide patients in either the usual care control group or the CardioCoach intervention group (clinical trial registration with www.clinicaltrials.gov; identifier NCT03294811). All patients provided written informed consent and were followed for 6 months after study enrolment. The study complies with the Declaration of Helsinki, and the study protocol was approved by the local committee on human research.

### Usual Care Control Group

ESC guidelines on uptitration of β-blocker and/or ACE-I therapy are primarily intended to be used by physicians. Therefore, medication dose adaptions were performed during occasional outpatient visits to the cardiologist or general practitioner. Medication doses were determined based on patient’s vital sign measurements, overall well-being, and symptoms. Besides an additional follow-up visit at 3 months, we did not modify the usual care as per standard practice organized in the institution where patients have a scheduled follow-up visit at 6 months.

### CardioCoach Intervention Group

Patients allocated to the CardioCoach intervention group also had a scheduled follow-up visit at 3 months and 6 months. For these patients, the usual care was supplemented with the CardioCoach follow-up tool to proactively uptitrate β-blocker and ACE-I treatment and improve medication adherence for β-blocker, ACE-I, and diuretic treatment. In terms of diuretic treatment, only medication adherence was monitored because it was not part of the active uptitration protocol. This intervention included a two-way communication platform connected to remote monitoring devices such as a weighing scale and blood pressure monitor to collect vital measurements (ie, weight, blood pressure, and heart rate), in which patients were followed by technical and clinical call centers. Medical follow-up (eg, medication uptitration, alerts on threshold crossing) was done by the clinical call center in the hospital, whereas technical follow-up (eg, missed transmissions, technical issues) was done by Remedus (Aartselaar, Belgium). Both call centers were active during working hours; notifications received during the weekend were read on Monday.

### The CardioCoach Follow-Up Tool for Semiautomatic Medication Uptitration

**Figure 1:**
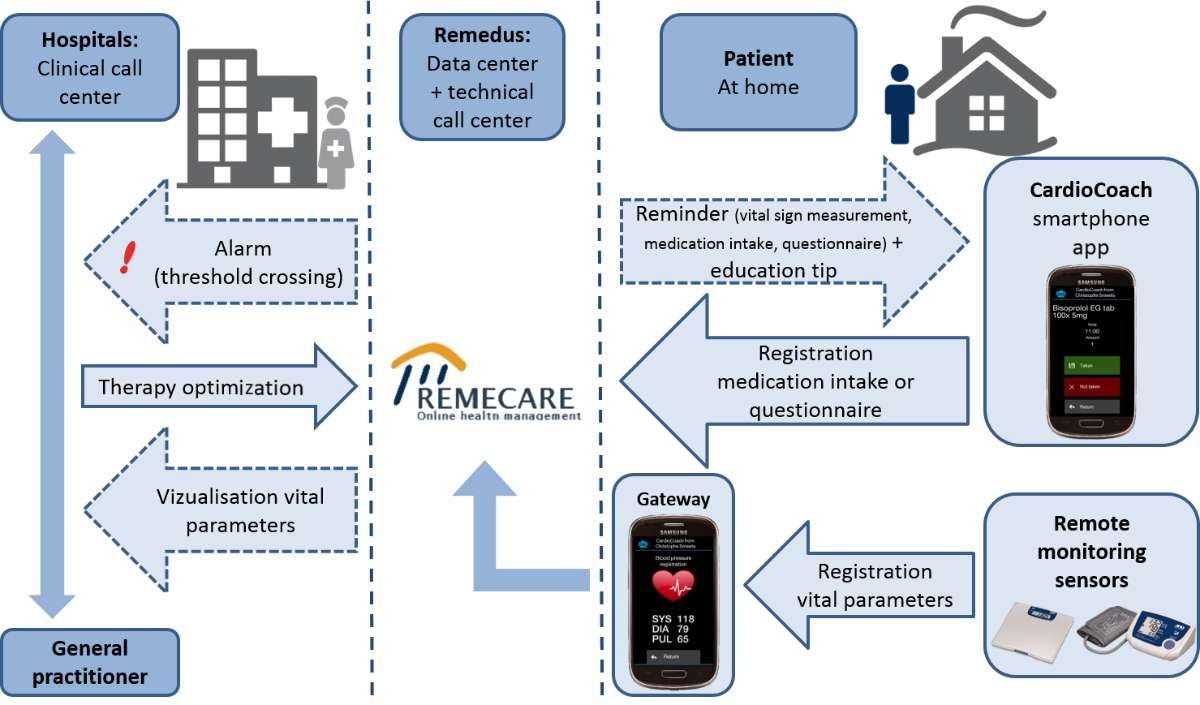
Overview of the CardioCoach follow-up tool.

The two-way communication platform consisted of a smartphone with the preinstalled CardioCoach app, a blood pressure monitor, weighing scale, and a Web-based health management server (Remecare) with a clinical dashboard for the care provider (HF nurse). An overview of the CardioCoach follow-up tool can be found in Figure 1.

The CardioCoach app was used to trigger the patient to conduct different actions, such as record a vital sign measurement, complete a questionnaire, and confirm medication intake by sending reminders at predefined time points. For each action, a 5-hour time window was set in which the patient could record all necessary data. This time window could be customized for each patient and was made available an hour before and 4 hours after the ideal recording time. All vital sign measurements were transmitted automatically to the CardioCoach app without manual patient input. The patient-specific medication scheme for β-blocker, ACE-I, and diuretic treatment was automatically uploaded to the patients’ smartphones every morning to inform them about their actual medication dose for that day. When changes were applied to the medication scheme, the patient was notified via a pop-up message, which he/she had to confirm. In addition, a daily education tip was pushed by the smartphone app to the patient covering different HF disease aspects (eg, tips to manage fluid and salt restriction, exercise). Screenshots of the CardioCoach app are shown in Multimedia Appendix 1.

All information gathered via the CardioCoach smartphone app (ie, vital signs, questionnaires, medication intake) was automatically transmitted to a secured Web-based health management server (ie, Remecare) without patient input. On this server, the completeness of patient data and possible deviations of vital signs based on predefined thresholds were verified.

When a patient does not record medication intake or vital sign data or does not complete a questionnaire, a pop-up message was pushed 2 hours after the ideal recording time via the smartphone to inform the patient about the missed registration and stress the importance of this information for the medication uptitration process. If the patient still did not complete the required action 4 hours after the ideal recording time, a *no registration* tag was recorded in the clinical database and the patient was contacted by the technical call center within 12 hours. In case of missed medication intake, the technical call center asked whether the patient forgot to register the medication intake or whether the patient forgot to take the required medication. In case of vital sign thresholds crossings for 3 consecutive days (Table 1), an automatic custom-made HF questionnaire was pushed to the patient via the CardioCoach smartphone app to gain insight about his/her general well-being or symptoms related to deviating vital signs, and a message was sent to the clinical call center to review the vital sign data and questionnaire (Multimedia Appendix 2).

### CardioCoach Medication Uptitration With Clinical Decision Support Algorithm

In the CardioCoach intervention group, β-blocker and ACE-I medication uptitration was supported by a clinical decision support algorithm, initiated at study inclusion. The algorithm generated a medication uptitration proposal at fixed moments in time during the first 3 months of follow-up, known as the active uptitration phase. Moreover, every 2 weeks, the algorithm alternately generated a medication uptitration proposal for either the β-blocker or ACE-I.

**Table 1:** Vital sign thresholds.

Parameter	Thresholds for 3 consecutive days
Weight	Baseline weight + 2 kg
Heart rate	<60 bpm^a^ or >100 bpm
Systolic blood pressure	<90 mm Hg or >160 mm Hg
Diastolic blood pressure	<60 mm Hg or >95 mm Hg

^a^bpm: beats per minute.

**Figure 2:**
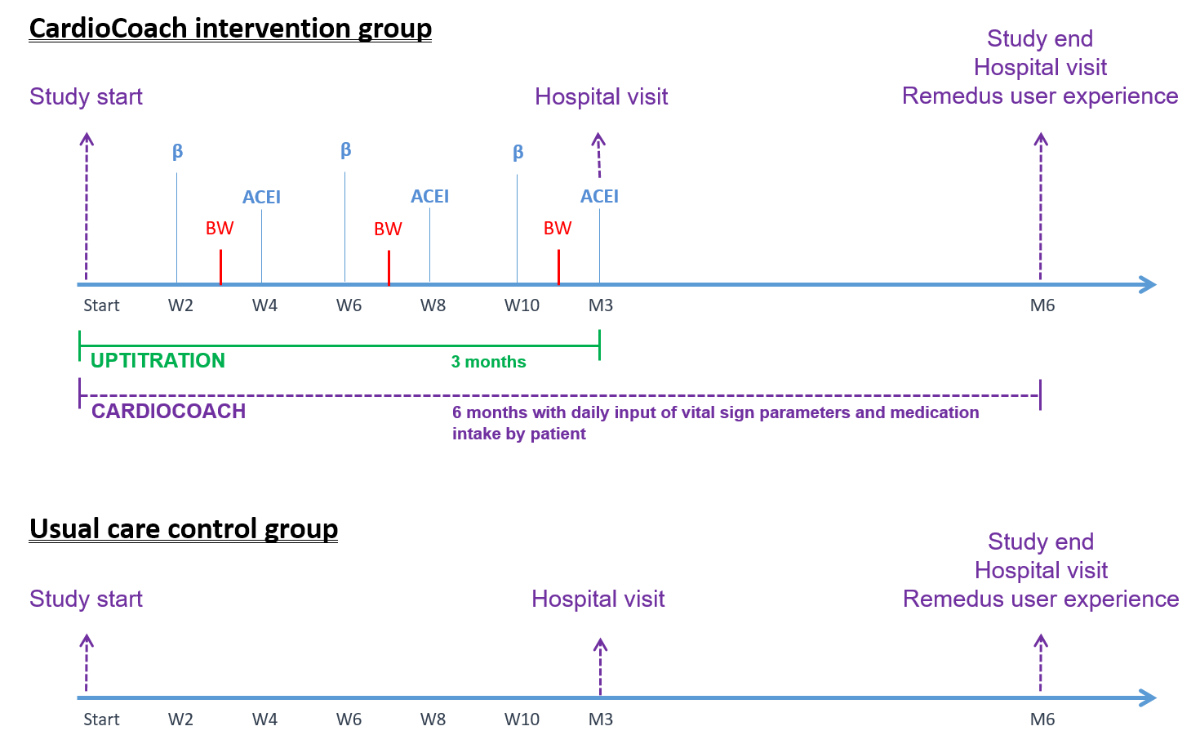
Overview of the study protocol for both the CardioCoach intervention group and usual care control group. ACE-I: angiotensin-converting enzyme inhibitors; BW: blood withdrawal.

At the beginning of week 2, the first proposal was generated, which comprised β-blocker uptitration, followed by another proposal for ACE-I uptitration at week 4. In total, there were 6 uptitration proposals during the first 3 months. Before each proposal, the short version of the custom-made HF questionnaire (Multimedia Appendix 2) was pushed to the patient’s smartphone to enquire about his/her general health and expose possible medication-induced side effects, which would help in deciding the safety of medication uptitration to the next level. A week before the ACE-I uptitration proposal, the algorithm generated a blood withdrawal request for analysis on kidney function (Figure 2). The type of proposal, generated by the algorithm, was generated based on predefined decision trees, taking into account all gathered patient information (vital signs, blood parameters, and questionnaires), which could include the following: (1) medication uptitration to the next level, (2) no uptitration or (3) medication uptitration to the next level only possible after evaluation by the HF nurse due to incomplete data, aberrant vital sign data, or aberrant blood parameters.

Before implementation of the updated medication scheme on the patient’s smartphone app, every proposal of the algorithm was reviewed by a dedicated HF nurse. The nurse could either choose to confirm the proposal, to call the patient before taking a decision, to make other changes to the patient’s medication scheme or to leave it unchanged, or indicate that the optimal medication dose had been reached. During the last 3 months of follow-up (ie, from 3 to 6 months), the active uptitration algorithm was deactivated and was followed by a less intensive follow-up phase during which medication intake and vital sign parameters were still monitored and medication uptitration on the discretion of the HF nurse could still proceed.

Finally, at 6 months of follow-up, patients from the CardioCoach intervention group were provided with a CardioCoach user experience questionnaire to gain feedback on the use of the CardioCoach smartphone app, the remote monitoring sensors, the contact with HF nurses and technical follow-up team.

**Table 2:** Maximum daily dose as recommended by European guidelines.

Active ingredient		Max daily dose (mg)
**ACE-I^a^**		
	Perindopril	10
	Enalapril	10
	Ramipril	10
	Lisinopril	20
	Candesartan	16
	Losartan	100
**β-blocker**		
	Bisoprolol	10
	Nebivolol	5

^a^ACE-I: angiotensin-converting enzyme inhibitors.

### Outcome Measurements

Outcome measures included CardioCoach user experience, (therapeutic) adherence, call center statistics, algorithm performance, and the number of patients on guideline-recommended medication dose for β-blocker and ACE-I (Table 2) at both 3 and 6 months of follow-up.

### Statistical Analysis

Demographic and functional characteristics were compared using descriptive statistics. Continuous variables were expressed as mean ± standard deviation if normally distributed, or otherwise as median (interquartile range, IQR). To define statistical differences between both groups, the independent samples student *t* test and Mann-Whitney *U* test were used for normally and not normally distributed continuous variables, respectively. The chi-square test and Fisher exact test were used accordingly for categorical variables. To define statistical differences between New York Heart Association class, the Kruskal-Wallis test was used. The significance level for tests was two-sided with an alpha of .05. All statistical analyses were performed using the Statistical Package for Social Sciences version 24.0 (IBM SPSS Inc, Chicago, Illinois, USA).

## Results

### Study Population

In total, 25 patients were included in the CardioCoach study. One patient dropped out before 3 months of follow-up and was therefore excluded from analysis. After 3 months of follow-up, 2 more patients dropped out but were still included in the analysis until 3 months of follow-up, because they completed the active medication uptitration phase. The final study population consisted of 24 patients: 14 patients were included in the CardioCoach intervention group and 10 patients were included in the usual care control group. Baseline characteristics of the study population at the time of inclusion are provided in Table 3. At the time of study enrollment, no significant between-group differences were observed in clinical characteristics or the use of medications commonly prescribed to patients with HF.

### CardioCoach Medication Uptitration With Clinical Decision Support Algorithm

On the basis of gathered data, the CardioCoach algorithm generated 72 medication uptitration proposals in total. In 7% (5/72) of the cases, the algorithm generated a conclusive proposal, whereas in 93% (67/72) of cases, the decision was left up to the HF nurse. This was mainly due to aberrant (67%, 48/72) or incomplete (25%, 18/72) data. Table 4 summarizes the frequency of the different algorithm uptitration proposals.

After each automatic uptitration proposal from the algorithm, the HF nurses in the clinical call center received a notification, which they had to consider. Nurses could respond in different ways to the uptitration proposal (Table 5). The algorithm proposal was confirmed by the HF nurse in 69% (50/72) of cases, and in 35% (25/72) of cases the patient was contacted for further interrogation before decision. No adverse events or false positive uptitration proposals were reported.

### Therapeutic Adherence

Overall, therapeutic adherence as confirmed by the patient via the smartphone app (8315/10,825, 76.81%) or via the technical call center after contacting the patient (1703/10,825, 15.73%) for the 3 drug treatments was 92.55% (10,018/10,825), with, respectively, 97.12% (3239/3335) for β-blockers, 94.89% (3549/3740) for ACE-I, and 86.13% (3230/3750) for diuretics. In 1 out of 5 cases, patients did not record medication intake into the CardioCoach smartphone app, and the technical call center had to contact the patients to verify medication intake (Table 6). In terms of vital sign registration, patient adherence was 94.66% (4504/4758). In 12.65% (602/4758) of these cases, technical issues hindered automatic transfer of vital sign data to the online database, and the technical call center had to contact the patient to receive the data, and in 5.34% (254/4758) no vital sign measurement was recorded (Table 6).

**Table 3:** Baseline characteristics of the study population at the moment of study inclusion (N=24). Continuous data are expressed as mean (SD) if normally distributed, and dichotomous data are expressed as n (%).

Variables		CardioCoach intervention group (n=14)	Usual care control group (n=10)	*P* value
Male gender, n (%)		9 (64)	6 (60)	>.99
Age, years, mean (SD)		63 (15)	60 (15)	.55
Body mass index, mean (SD)		28 (5)	28 (5)	.88
Heart rate, mean (SD)		73 (13)	73 (13)	.99
Systolic blood pressure, mean (SD)		112 (14)	127 (25)	.08
Diastolic blood pressure, mean (SD)		75 (12)	75 (12)	.98
New York Heart Association functional class (II/III), n (%)		6 (43)/6 (43)	4 (40)/5 (50)	.92
Left ventricular ejection fraction percentage, mean (SD)		28 (7)	29 (7)	.84
QRS width, ms, mean (IQR^a^)		100 (90-121)	100 (92-121)	.89
Ischemic cardiomyopathy, n (%)		4 (29)	1 (10)	.36
Dilated cardiomyopathy, n (%)		5 (36)	5 (50)	.68
**Risk factors and comorbidities, n (%)**				
	Obesity	9 (64)	3 (30)	.10
	Arterial hypertension	9 (64)	3 (30)	.10
	Smoking	9 (64)	9 (90)	.34
	Family history of cardiovascular diseases	7 (50)	4 (40)	.70
	Hypercholesterolemia	9 (64)	5 (50)	.68
	Chronic kidney disease	2 (14)	0 (0)	.49
	Atrial fibrillation	6 (43)	4 (40)	>.99
	Diabetes	3 (21)	1 (10)	.62
	Chronic obstructive pulmonary disease	1 (7)	1 (10)	>.99
Pro-Brain Natriuretic Peptide, mean (IQR)		559 (118-1278)	262 (129-467)	.44
Estimated glomerular filtration rat, mean (SD)		50 (28)	65 (19)	.16
**Medication use, n (%)**				
	Angiotensin converting enzyme inhibitor	7 (50)	3 (30)	.42
	β-blocker	7 (50)	3 (30)	.42
	Spironolactone	1 (7)	1 (10)	>.99
	Loop diuretic	1 (7)	2 (20)	.39
	Statin	7 (50)	3 (30)	.42
	Calcium channel blockers	0 (0)	1 (10)	.42
	Antidiabetic medication	1 (7)	1 (10)	>.99
**Technological experience, n (%)**				
	Normal cell phone	8 (57)	6 (60)	>.99
	Smartphone	3 (21)	3 (30)	.67
	Computer at home	7 (50)	4 (40)	.70
	Internet connection at home	2 (14)	3 (30)	.62
	Tablet at home	7 (50)	4 (40)	.70

^a^IQR: interquartile range.

**Table 4:** Overview of the different algorithm uptitration proposals and their frequency.

Type of uptitration proposal	Full sample (N=72), n (%)	β-blocker group (n=41), n (%)	ACE-I^a^ group (n=31), n (%)
Uptitration to next level	1 (1)	1 (2)	0 (0)
No uptitration to next level	4 (6)	3 (7)	1 (3)
Uptitration dependent on evaluation by heart failure nurse, due to incomplete data	18 (25)	11 (27)	7 (23)
Uptitration dependent on evaluation by heart failure nurse, due to aberrant data	48 (67)	26 (63)	22 (71)
Uptitration dependent on evaluation by heart failure nurse, due aberrant blood parameters	1 (1)	0 (0)	1 (3)

^a^ACE-I: angiotensin-converting enzyme inhibitors.

**Table 5:** Overview of the different responses of the heart failure nurses to the algorithm uptitration proposals.

Response of nurses to uptitration proposal	Full sample (N=72), n (%)	β-blocker group (n=41), n (%)	ACE-I^a^ group (n=31), n (%)
Confirm algorithm proposal	50 (69)	29 (71)	21 (68)
Patient was contacted before decision was made	25 (35)	17 (41)	8 (26)
Change of other medication	10 (14)	8 (20)	2 (6)
Optimal medication dose reached	25 (36)	13 (32)	12 (39)

^a^ACE-I: angiotensin-converting enzyme inhibitors.

**Table 6:** Therapeutic adherence for medication intake and vital sign measurement recording.

Therapeutic adherence		n (%)	
**Medication intake (N=10,825)**			
	Confirm via smartphone		8315 (76.81)
	Confirmed via Remedus		1703 (15.73)
	Declined via smartphone		351 (3.24)
	Declined via Remedus		456 (4.21)
**Vital sign measurement (N=4758)**			
	Confirm via smartphone		3902 (82.00)
	Confirmed via Remedus		602 (12.65)
	No recording		254 (5.34)

### Technical Call Center Statistics

For the 14 CardioCoach patients, the Remedus call center made 831 phone calls in total, with a median of 41 phone calls per patient (IQR 32-65). Phone calls were initiated in case of missed vital sign measurements (n=136), missed medication intake (n=661; diuretic intake 44.0% [291/661], ACE-I intake 34.9% [231/661], and β-blocker intake 21.0% [139/661]), or missing questionnaires (n=34). Due to the limited technical skills of the study participants, technical problems could hardly be solved remotely, and therefore, a device swap was performed in 10 patients: 4 patients had 1 device swap, 5 patients had 2 device swaps, and 1 patient had 3 device swaps.

### CardioCoach User Experience

Among the CardioCoach user experience questionnaire, 4 questionnaires were missing: 3 due to early study termination and 1 due to an issue with the Web-based questionnaire platform. Detailed results of these questionnaires can be found in Multimedia Appendices 3-5. In general, patients were very satisfied, and mentioned the ease of use of the smartphone app and remote monitoring sensors. Daily coaching tips were reviewed as being positive and stimulating. In addition, patients experienced an extra sense of safety, and 50% of patients were eager to continue using the CardioCoach follow-up tool after the study ended. Due to the CardioCoach app, 80% of patients reported an increased medication adherence. Patients reported a positive experience in terms of communication with both the technical and clinical call centers. Interestingly, patients were indifferent about the fact that their parameters were being reviewed by an external, home nursing company. Finally, patients did mention a large number of technical issues (eg, connectivity issues, problems with the remote monitoring sensors).

**Figure 3:**
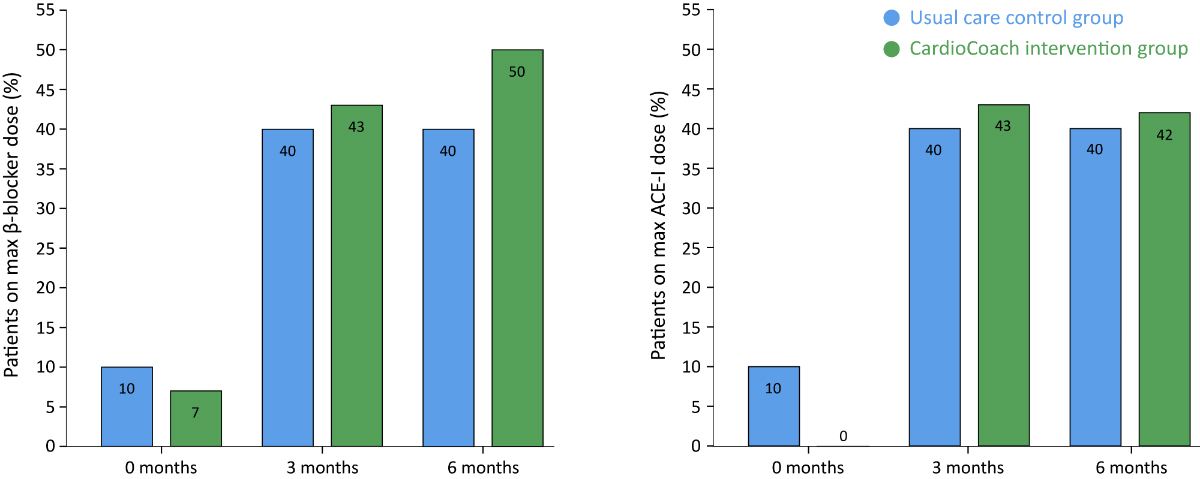
The number of patients on maximum daily dose as recommended by European Society of Cardiology guidelines for both β-blockers (left) and ACE-I (right). No significant differences were observed between both groups. ACE-I: angiotensin-converting enzyme inhibitors.

### Medication Uptitration

No significant differences were observed in the number of patients on guideline-recommended maximum β-blocker dose in the CardioCoach intervention group when compared with the usual care control group at both 3 months (43% vs 40%, *P*>.99) and 6 months (50% vs 40%, *P*=.69) of follow-up (Figure 3).

Additionally, in terms of ACE-I uptitration, no significant differences were observed at both 3 months (43% vs 40%, *P*>.99) and 6 months (42% vs 40%, *P*>.99) of follow-up (Figure 3). In addition, there was no difference in terms of time taken to uptitrate to guideline-recommended medication dose. All patients who reached the guideline-recommended dose did so before 3 months of follow-up (except 1 case for β-blockers).

## Discussion

### Principal Findings

Since 1997, ESC guidelines for the diagnosis and treatment of acute and chronic HF have recommended the optimization of drug treatment as the first step in patients diagnosed with HF [1,27]. Unfortunately, these guidelines are insufficiently implemented in clinical practice and many HF patients are still on suboptimal medication dose [15,18-20]. This paper describes the rationale and feasibility of a novel two-way communication platform with decision support algorithms, in combination with a smartphone app, blood pressure monitor, and weighing scale, intended to support β-blocker and ACE-I uptitration remotely. The success rate of studies monitoring weight, blood pressure, and heart rate to improve clinical outcome is rather low, probably because they are unable to capture the complexity of HF disease progression, which often involves multiple comorbidities [28-33]. However, the benefits of monitoring weight, blood pressure, and heart rate for medication uptitration have only recently been studied [21,26].

The results of this feasibility study with 24 patients, monitored for a period of 6 months, showed a marginal increase in the number of patients on guideline-recommended β-blocker and ACE-I dose when using the CardioCoach remote monitoring follow-up tool compared with usual care alone. However, in comparison with previous studies, both our intervention and control group consisted of a higher number of patients, who were on guideline-recommended medication dose. Maggioni et al [18] and Heywood et al [19] reported, respectively, 29% and 35% of patients on target dose for ACE-I and 17% and 15% for β-blockers. This suggests that the usual care provided in our institution is superior to the standard care described in literature, and the addition of the CardioCoach follow-up tool can lead to comparable and even slightly better results. Hence, remote monitoring could be a suitable method for increasing the number of HF patients on guideline recommended target dose, especially in centers with less intensive usual care follow-up.

Feedback received from the patients using the CardioCoach follow-up tool revealed overall good patient satisfaction in terms of both the use of the remote monitoring devices and the contact between the patient and technical and clinical call centers. This resulted in excellent overall therapeutic adherence of the patients during the entire study period for medication intake (92.55%, 10,018/10,825) and vital sign measurements (94.66%, 4504/4758). In spite of the frequent reminders via the smartphone, the CardioCoach follow-up tool was well accepted by the patients as compared with remote monitoring strategies used in previous studies [34,35]. Unfortunately, patients did mention many technical issues, which are deduced from the large number of phone calls between the patient and the technical call center of Remedus. In 1 out of 5 cases, patients were contacted by the technical call center to verify medication intake. In most of these cases, the patients confirmed medication intake, but due to the technical issues, this information was not transmitted to the Remecare platform. Only 7.45% (807/10,825) of cases reported that the patient had not taken his/her medication. This was rarely due to the forgetfulness of the patient, but mostly because of a change in patient’s medication scheme outside the CardioCoach environment (eg, by a general practitioner). In terms of vital sign measurements, 12.65% (602/4758) of the measurements were collected over the phone by the technical call center as technical issues hindered automatic transfer of vital sign data to the Remecare Platform. These technical issues also included issues that arose because of the technophobe elderly study population (eg, problems changing/charging device batteries, reboot smartphone). In 5.34% (254/4758) of cases, defective remote monitoring sensors made it impossible to record a vital sign measurement. The high number of technical issues clearly demonstrates the need for a separate technical call center to handle these issues, avoid extra work burden for the clinical call center, and ensure complete data for clinical decision making. Although the next generation of seniors will probably be more familiar with technical developments, technical improvements are still necessary to further decrease these issues.

In this study, the algorithm was built with a large safety margin to avoid false positive uptitration proposals, which has led to a low number of conclusive proposal by the algorithm (7%, 5/72). In addition, every proposal had to be validated by a dedicated HF nurse. In 69% (50/72) of the cases, the HF nurse confirmed the algorithm proposal. This shows that parameter thresholds can be confined. In this sense, the current feasibility study was very useful for the future development and improvement of an optimal two-way communication system between patients and caregivers. On the basis of feedback from both patients and HF nurses, improvements can be made to the next generation, which will take into account the work efficiency of the HF nurses and enable a customized approach for patients (eg, patient-specific or less confined parameter thresholds, patient-specific uptitration scheme). The CardioCoach follow-up tool is very efficient in facilitating information exchange between the different care providers (ie, HF specialist, HF nurse, general practitioner, home nurse) and enables a safe way for medication uptitration, as there were no adverse events or false positive uptitration proposals reported. The use of the CardioCoach follow-up tool has been shown to be feasible when combined with a technical call center to handle technical issues and reduce the workload of the clinical call center. This study was unable to demonstrate a significant improvement of the CardioCoach follow-up tool on the number of patients on maximum guideline-recommended β-blocker and ACE-I dose. Probably, this is related to the fact that patients in the control group were also enrolled in a dedicated HF outpatient disease management program, where HF medication dosages were being optimized by intensive follow-up by specialized HF nurses and HF specialists. Hence, the CardioCoach follow-up tool might be more suitable in centers with less intensive HF disease management programs.

### Study Limitations

This feasibility study should be interpreted in the light of some limitations to place the study findings into a correct context. First, the small sample size and the single-center character may impact its external validity. Therefore, these results should be interpreted as hypothesis generating, and an additional multi-center study is necessary to confirm these results. In this study, the control group received the usual care as per standard practice organized in the institution and received no remote monitoring sensors. This is a general issue in multiple remote monitoring studies, which should be taken into account when interpreting study findings as relevant information from the control group may be missing. An alternative control group could be a group in the same setting (ie, with remote monitoring sensors), but without a physician reviewing the data. Next, technical improvements (eg, Bluetooth connectivity, battery autonomy) are necessary to improve the efficiency of the CardioCoach follow-up tool. Finally, the patient population used to conduct the feasibility study was recruited in a tertiary care center with a specialized HF clinic. Due to the high quality of the usual care provided (reflected by the high number of patients on maximum guideline-recommended medication dose in the usual care group compared with literature) with intensive outpatient follow-up, the institution under study may not have been the optimal choice to demonstrate a potential benefit of the CardioCoach follow-up tool on medication uptitration.

### Conclusions

This study shows the feasibility and safety of a novel two-way communication platform with decision support algorithms in combination with remote monitoring sensors in implementing guideline recommendations concerning β-blocker and ACE-I uptitration. In addition, the CardioCoach follow-up tool was found to be efficient in facilitating information exchange and improving coordination among different care providers. Patients’ satisfaction was reported to be high, which has led to excellent adherence rates during a relative long follow-up period of 6 months. Many technical issues arose, clearly indicating the need for a technical call center. A larger multicenter randomized controlled trial needs to be conducted in centers with minimal usual care follow-up to assess the potential benefits of guideline-recommended medication dose.

## Appendix

Multimedia Appendix 1 Screenshots from the CardioCoach smartphone app with from top to bottom registration of medication intake, weight, blood pressure, and heart failure questionnaire.

Multimedia Appendix 2 Custom-made heart failure questionnaire pushed to the patient before an uptitration proposal or in case of aberrant vital sign data.

Multimedia Appendix 3 Detailed results of the CardioCoach user experience questionnaire (Part 1).

Multimedia Appendix 4 Detailed results of the CardioCoach user experience questionnaire (Part 2).

Multimedia Appendix 5 Detailed results of the CardioCoach user experience questionnaire (Part 3).

Multimedia Appendix 6 CONSORT‐EHEALTH checklist (V 1.6.1).

## Abbreviations

ACE-I: angiotensin-converting enzyme inhibitors
ESC: European Society of Cardiology
HF: heart failure
IQR: interquartile range
