# Supplementary figures and images for: A Novel Intelligent Two-Way Communication System for Remote Heart Failure Medication Uptitration (the CardioCoach Study): Randomized Controlled Feasibility Trial
Source: JMIR Cardio. 2018 Apr 4;2(1):e8. doi: 10.2196/cardio.9153 (PMC6834244; doi:10.2196/cardio.9153)

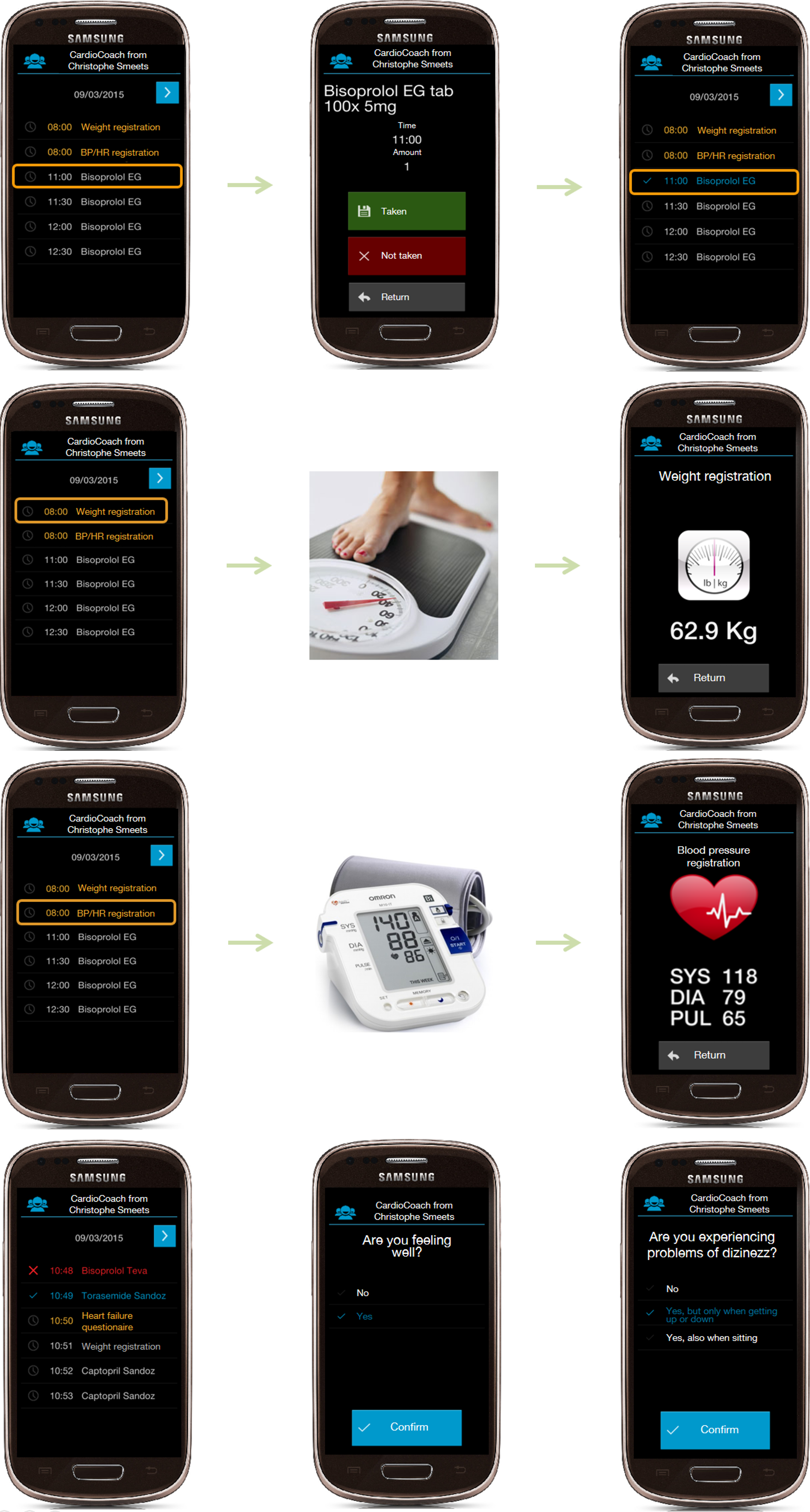

Supplement: Multimedia Appendix 1 [file cardio_v2i1e8_app1.png]

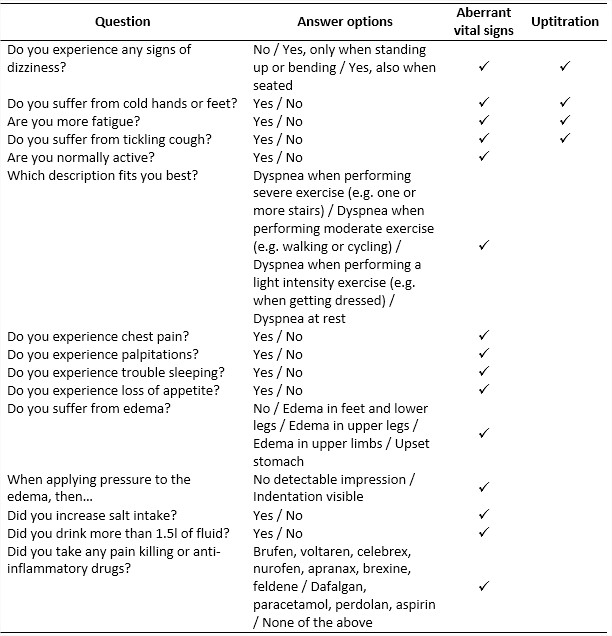

Supplement: Multimedia Appendix 2 [file cardio_v2i1e8_app2.jpg]

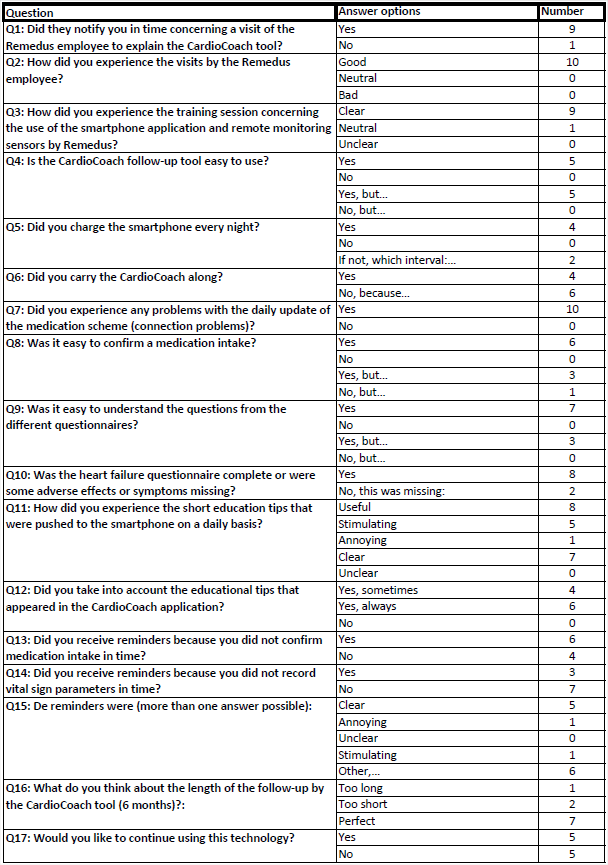

Supplement: Multimedia Appendix 3 [file cardio_v2i1e8_app3.png]

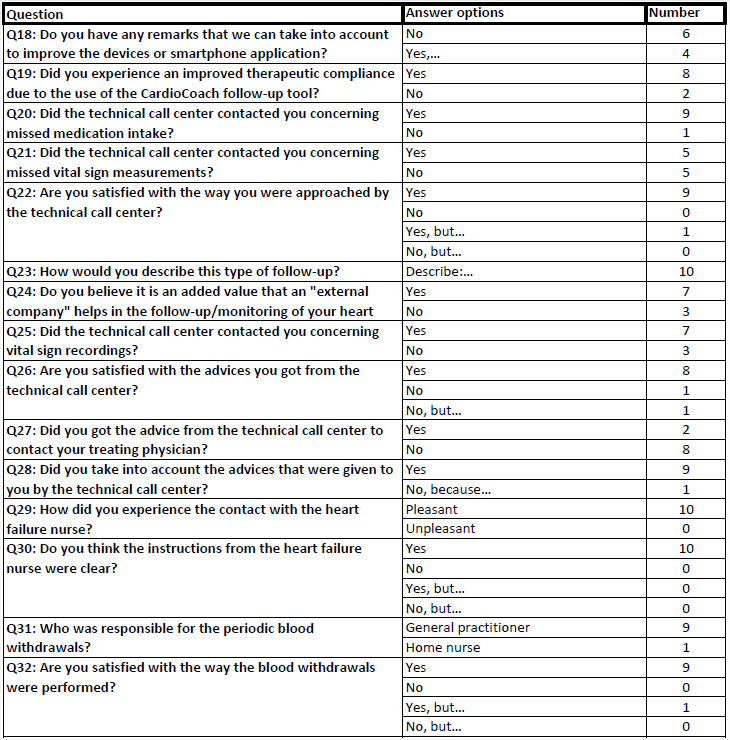

Supplement: Multimedia Appendix 4 [file cardio_v2i1e8_app4.png]

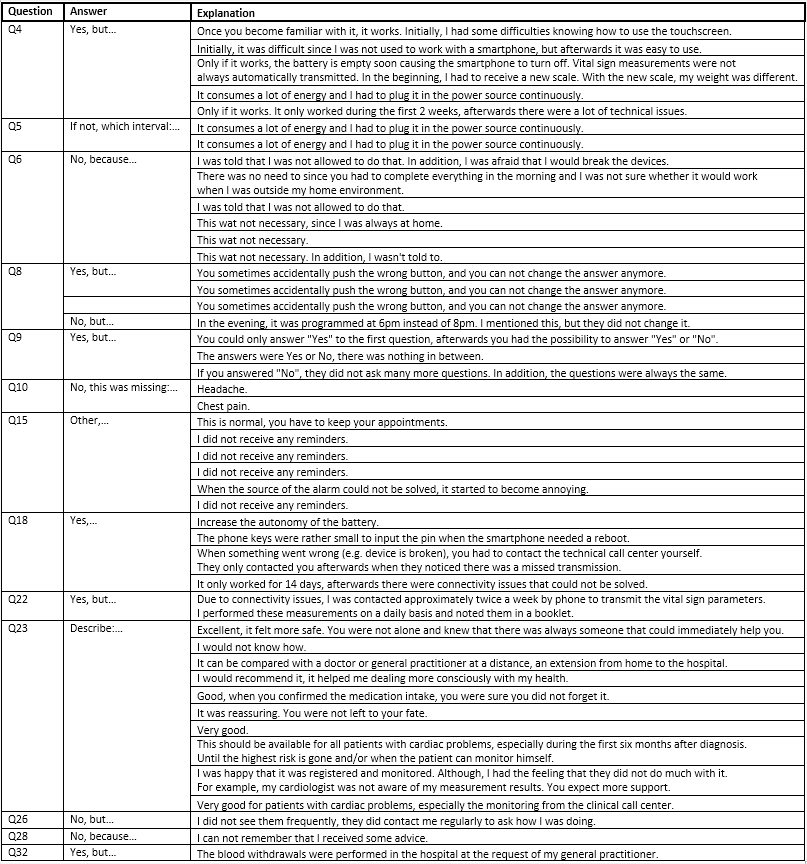

Supplement: Multimedia Appendix 5 [file cardio_v2i1e8_app5.png]
